# Most bowel cancer symptoms do not indicate colorectal cancer and polyps: a systematic review

**DOI:** 10.1186/1471-230X-11-65

**Published:** 2011-05-30

**Authors:** Barbara-Ann Adelstein, Petra Macaskill, Siew F Chan, Peter H Katelaris, Les Irwig

**Affiliations:** 1 Prince of Wales Clinical School, Faculty of Medicine, University of NSW, Sydney, Australia; 2 associate professor, STEP, School of Public Health, University of Sydney, Australia; 3 biostatistician, STEP, School of Public Health, University of Sydney, Australia; 4 gastroenterologist, Concord Repatriation General Hospital, University of Sydney, Australia; 5 professor of epidemiology, STEP, School of Public Health, University of Sydney, Australia; 6 Screening and Test Evaluation (STEP) Program, School of Public Health, University of Sydney, NSW 2006, Australia

**Keywords:** colorectal cancer, colorectal polyps, bowel symptoms, rectal bleeding, change in bowel habit

## Abstract

**Background:**

Bowel symptoms are often considered an indication to perform colonoscopy to identify or rule out colorectal cancer or precancerous polyps. Investigation of bowel symptoms for this purpose is recommended by numerous clinical guidelines. However, the evidence for this practice is unclear. The objective of this study is to systematically review the evidence about the association between bowel symptoms and colorectal cancer or polyps.

**Methods:**

We searched the literature extensively up to December 2008, using MEDLINE and EMBASE and following references. For inclusion in the review, papers from cross sectional, case control and cohort studies had to provide a 2×2 table of symptoms by diagnosis (colorectal cancer or polyps) or sufficient data from which that table could be constructed. The search procedure, quality appraisal, and data extraction was done twice, with disagreements resolved with another reviewer. Summary ROC analysis was used to assess the diagnostic performance of symptoms to detect colorectal cancer and polyps.

**Results:**

Colorectal cancer was associated with rectal bleeding (AUC 0.66; LR+ 1.9; LR- 0.7) and weight loss (AUC 0.67, LR+ 2.5, LR- 0.9). Neither of these symptoms was associated with the presence of polyps. There was no significant association of colorectal cancer or polyps with change in bowel habit, constipation, diarrhoea or abdominal pain. Neither the clinical setting (primary or specialist care) nor study type was associated with accuracy.

Most studies had methodological flaws. There was no consistency in the way symptoms were elicited or interpreted in the studies.

**Conclusions:**

Current evidence suggests that the common practice of performing colonoscopies to identify cancers in people with bowel symptoms is warranted only for rectal bleeding and the general symptom of weight loss. Bodies preparing guidelines for clinicians and consumers to improve early detection of colorectal cancer need to take into account the limited value of symptoms.

## Background

Adenocarcinoma of the colon and rectum is the third most common cancer and the third leading cause of death in the United Kingdom. In 2004, there were almost 40,000 new cases diagnosed, and about 16,000 deaths from colorectal cancer [1]. The symptoms with which colorectal cancer are purported to present most commonly include alteration in bowel habit, rectal bleeding, abdominal pain and weight loss [2,3].

Bowel symptoms occur commonly in the community and are often self limiting. There is little information available about why or when people seek medical attention for them [4,5]. Colonoscopies to exclude colorectal cancer are done frequently for a wide range of bowel symptoms, a practice suggested by guidelines [6] although it is unclear which symptoms, if any, improve the diagnostic yield of cancers or polyps. The costs, both clinical and financial, of performing colonoscopies are high. To inform policy, it is therefore important to assess which symptoms, if any, are associated with cancer and precancerous polyps.

The aim of this systematic review was to assess the evidence about the association between bowel symptoms and colorectal cancer or colorectal polyps.

## Methods

### Search strategy

We conducted a comprehensive search of the health literature for all studies evaluating symptoms and colorectal cancer or polyps. The search was undertaken in December 2008 to identify relevant studies. We searched MEDLINE (1966-2008) and complete EMBASE, using a list of symptoms and diagnoses as MeSH headings; the full search strategy is given in Additional File 1. The literature selection was based initially on the paper title, and if thought relevant, followed in turn by the abstract or full paper. Foreign language papers were translated into English. The literature search and selection of papers for full review, was carried out independently on two occasions 6 months apart by one reviewer.

Further, the references included in all selected papers, as well as review articles, were assessed for possible inclusion in the systematic review. The citations for each paper identified for inclusion in the review were also checked using the cited reference component of the Web of Science database.

### Inclusion criteria

For inclusion in the review, papers had to provide sufficient data about both the symptom and diagnosis (colorectal cancer or polyps) and provide a 2×2 table of symptoms by diagnosis, or the data from which that table could be constructed. We did not restrict included papers to certain study types, and we have extensively explored whether study characteristics had any effect on the findings. Papers (n = 8) that did not differentiate between cancers and polyps were excluded. Only full papers were included: where a relevant conference abstract was found, the literature was searched for a more detailed description of the study. If no full paper was found, the abstract was not included (this occurred with 1 abstract) [7].

### Data extraction and methodological assessment

Extraction of data was performed by one reviewer (BA), with the complete set of data extracted independently on two separate occasions 6 months apart. Issues of uncertainty or discrepancy between the data extraction sets were referred to a second reviewer (LI); this occurred in 50% of papers. Agreement was subsequently obtained at consensus meetings.

For each study, data about methodology, quality and population characteristics were extracted. Items assessed included the clinical setting of the study, whether all participants had at least one symptom or some were asymptomatic (population type), whether each participant could have only one or more than one symptom reported, and study design items (patient recruitment from general or specialist practice settings, prospective or retrospective data collection, year of publication, consecutive patient recruitment, study type, reference standard used), and the ease with which data could be extracted from the paper. We also assessed the prevalence of cancer in each paper. The data categories and the assumptions required to extract the data are shown in Additional File 2.

One paper described two studies, for which we combined the data [8]. In the same paper, there were 'don't know' responses that were categorised as "present" for our analysis. This did not occur in more than 4.4% of responses.

We used colorectal cancer and polyps only as the two main outcome measures. Colorectal cancer included colon and rectal cancer, and included cancers that were confirmed by histology, as well as cancers listed as such in the papers but with no criteria given for the diagnosis. In general, papers providing information about polyps did not differentiate between polyps greater or less than 10 mm, or between different polyp histology; results are therefore for all polyps.

We have presented results for all symptoms for cancer, but for polyps and for comparisons between cancer and polyps we have included results only for those symptoms which showed a significant association for cancer.

### Statistical method

The estimated sensitivity and specificity were used to estimate the diagnostic odds ratio (DOR (=[sensitivity/(1-sensitivity)]/[(1-specificity)/specificity]) which provides a single summary measure of test accuracy for each study. A high DOR indicates high test accuracy; a test that performs no better than chance in discriminating between diseased and non-diseased persons has a DOR of one. Summary ROC (SROC) methods were used to investigate the accuracy of symptoms for the diagnosis of colorectal cancer (or polyps); and to investigate whether study methodology, quality and population characteristics were associated with the diagnostic performance of symptoms. Preliminary exploratory analyses for each symptom were conducted using the SROC linear regression method of Moses and Littenberg [9]. The log_e_(DOR) was modeled (using unweighted least squares) as a function of the underlying test positivity rate (logit (sensitivity) + logit(1-specificity)) which is a proxy for test threshold. Study and patient characteristics were fitted as covariates. Regression diagnostics were examined to identify outliers and potentially influential studies in determining the shape and position (accuracy) of the SROC curve.

Studies were further analysed using the hierarchical SROC (HSROC) model of Rutter and Gatsonis [10,11]. This mixed model is more complex, but more rigorous that the Moses and Littenberg method because it takes separate account of the uncertainty in the estimates of sensitivity and specificity within each study, and includes random study effects for both test accuracy and positivity criterion (proxy for threshold), thereby taking account of unexplained heterogeneity between studies. The model also allows test accuracy to vary with "threshold" through the inclusion of a scale (shape) parameter fitted as a fixed effect which provides for asymmetry in the SROC. Fixed effect covariates were fitted to assess whether accuracy, positivity criterion or the shape of the SROC was associated with study or patient characteristics. Empirical Bayes estimates of model parameters were obtained using PROC NLMIXED in SAS [12]. These parameter estimates were used to obtain summary estimates and 95% confidence intervals for sensitivity and specificity (summary operating point), and likelihood ratios. The area under the SROC curve (AUC) was computed using numerical integration. Where the summary curve was symmetric, the DOR is also reported as it is constant across all thresholds.

Covariates that showed at least very weak association (p < 0.25) with diagnostic performance in the preliminary analysis were included in the model to assess whether test accuracy, the positivity criterion and/or the shape of the SROC varied with population and study design characteristics. The chosen level for statistical significance was 5% (two sided). Where summary ROC curves being compared had the same shape, the relative DOR (RDOR) was used as the summary measure of the relative diagnostic performance, otherwise the AUC was used. Only results that were robust to the removal of an influential study are reported.

Comparison of the accuracy of each symptom for the diagnosis of colorectal cancer versus polyps was restricted to studies that provided data for both outcomes. This "paired" analysis, where the diagnosis is fitted as a covariate in the HSROC model, ensures that the comparison is not confounded by study or patient characteristics.

The fitted summary ROC curves derived from the HSROC model are displayed in ROC space, and are superimposed on the study specific estimates of sensitivity and specificity that are denoted by an ellipse. The horizontal and vertical dimensions of each ellipse are proportional to the square root of the number of non-diseased and diseased respectively. A cross is used to show the summary estimate of sensitivity and specificity. For the "paired" analyses, the two points for each study are joined by a line; points are denoted by circles that do not vary in size for the sake of clarity for these plots.

### Ethics approval

As this is a systematic review conducted on previously published papers and did not use patient level data, no approval was required.

## Results

The literature search yielded 14,121 articles, of which 248 were selected for full review. Three non-English papers were translated but only one met the inclusion criteria. We identified 62 eligible papers that provided relevant information separately for cancers and polyps [8,13-73]. Studies were published between 1960 and 2008. Quality and study characteristics and descriptors are shown in Additional File 2.

There was a wide range of symptoms included in the papers, with many papers providing information on several symptoms: 26 separate symptoms were included, as well as 3 combinations of symptoms (for example, bleeding together with change in bowel habit). In addition, some papers provided information about descriptions of bleeding. A full list of papers, with all outcomes, and symptoms is provided in Additional File 3.

The most commonly reported bowel symptoms were bleeding, change in bowel habit, constipation, diarrhoea and abdominal pain. Weight loss was the most common general symptom reported. Overall, only bleeding and weight loss showed any significant association with cancer (Table 1).

**Table 1:** Overview of results: Symptoms association with cancer.

Symptom	DOR* (95% CI*)	AUC**	Sensitivity (95% CI)	1-specificity (95% CI)	LR+ (95% CI)	LR- (95% CI)
Rectal bleeding^#^	2.6 (1.9-3.6) p < 0.001	0.66	0.46 (0.38-0.55)	0.25 (0.19-0.31).	1.9 (1.5-2.3)	0.7 (0.6-0.8)
*Blood mixed with stool: *	*3.1* *(2.0-4.8)* *p < 0.001*	*0.68*	*0.49* *(0.30-0.69)*	*0.24* *(0.13-0.40)*	*2.1* *(1.5-2.8)*	*0.7* *(0.5-0.9)*
*Blood: dark red*	*3.9* *(1.7 - 9.2)* *P = 0.004*	*0.71*	*0.29* *(0.09-0.65)*	*0.10* *(0.03-0.28)*	*3.1* *(1.6-6.0)*	*0.8* *(0.6-1.1)*
Change in bowel habit	1.5 (0.8-2.8) p = 0.16	0.57	0.32 (0.21-0.46)	0.24 (0.15-0.35)	1.4 (0.9-2.1)	0.9 (0.7-1.1)
Constipation	1.1 (0.8-1.5) p = 0.48	0.52	0.12 (0.08-0.18)	0.11 (0.07-0.16).	1.1 (0.8-1.5)	1.0 (1.0-1.0)
Diarrhoea	0.9 (0.4-1.7) p = 0.65	0.47	0.15 (0.07-0.28)	0.17 (0.09-0.29)	0.9 (0.5-1.6)	1.0 (0.9-1.1)
Abdominal pain	0.7 (0.5-1.1) p = 0.12	0.45	0.19 (0.13-0.28)	0.24 (0.17-0.33).	0.8 (0.6-1.1)	1.1 (1.0-1.2)
Weight loss	2.9 (1.6-5.0) p = 0.001	0.67	0.20 (0.12-0.31)	0.08 (0.05-0.13).	2.5 (1.5-4.0)	0.9 (0.8-1.0)

*DOR = diagnostic odds ratio. No association between symptom and cancer if DOR = 1

**AUC = area under the Receiver operating characteristic curve. No association between symptom and cancer if AUC = 0.5

# Bleeding of any type

Note: Results for bleeding types for which there 3 or more papers or for which the DOR was > 1 are shown. Results for other bleeding types are shown in Additional File 4.

Results are presented below for the association between each symptom and cancer. Where the association was significant, results are also presented for the association between that symptom and polyps.

### Rectal Bleeding

Forty papers provided information about the relationship between bleeding (of any type) and colorectal cancer [13-21,23-28,30,32,33,35,37,41,42,45-47,50,51,54-56,60-66,69,72,73], and 23 of these papers [13-17,20,21,23,24,26,28,30,33,41,42,46,47,54,56,61,63,72,73] also provided information about the relationship between bleeding and polyps.

**Figure 1:**
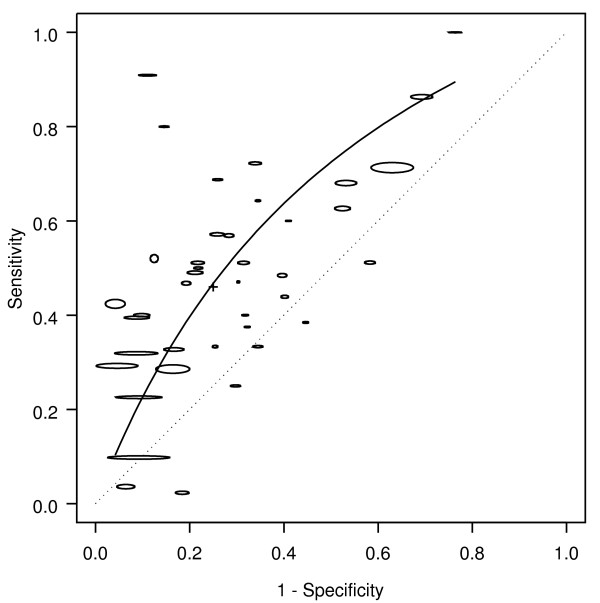
**Bleeding: SROC for cancer**. SROC = Summary ROC crc = colorectal cancer. The horizontal and vertical dimensions of the each ellipse are proportional to the square root of the number of non-diseased and diseased respectively. + shows the expected sensitivity and 1-specificity for the curve. The diagonal line joining (0,0) and (1,1) represents an ROC of no diagnostic value.

Rectal bleeding was associated with colorectal cancer (Figure 1). Based on all 40 studies, the AUC was 0.66, corresponding to a DOR of 2.6 (Table 1). Based on the summary operating point (shown by a cross on the figures), bleeding occurred in about half the patients with cancer (sensitivity 0.46), but also occurred in about a quarter of patients without cancer (1-specificity 0.25). Hence, the likelihood of cancer is approximately doubled in people presenting with bleeding (LR+ = 1.9): even if cancer is present in as many as 5% of people asked about symptoms, only 9% of those with rectal bleeding will have cancer. The corresponding likelihood of cancer in people presenting with no bleeding was LR- = 0.7.

The methodology, quality and population characteristics of the studies also influenced how bleeding was associated with cancer. The association of bleeding with colorectal cancer was higher (RDOR 2.2, 95% CI 1.2 - 4.0) when both symptomatic and asymptomatic people (AUC 0.71), rather than just those with symptoms (AUC 0.59) were included in the population from which patients were recruited. The accuracy of bleeding in diagnosing colorectal cancer was higher when colonoscopy (AUC 0.68), compared to all other diagnostic modalities (AUC 0.63), was used as the reference standard.

**Figure 2:**
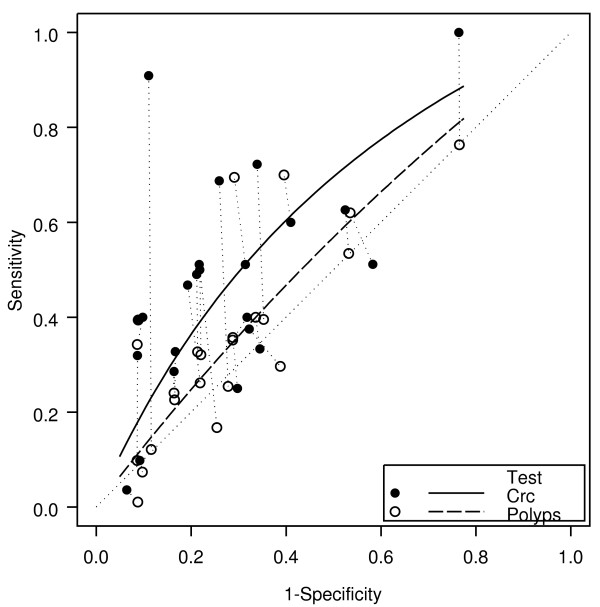
**Bleeding: cancer and polyp comparison**. crc = colorectal cancer. The horizontal and vertical dimensions of the each ellipse are proportional to the square root of the number of non-diseased and diseased respectively. + shows the expected sensitivity and 1-specificity for the curve. The paired points (one black and one open joined by a dotted line) represent a within study comparison. The diagonal line joining (0,0) and (1,1) represents an ROC of no diagnostic value.

Of the 23 "paired" studies that provided information about bleeding for both cancers and polyps, 18 had a higher DOR for cancer than for polyps (Figure 2), resulting in a relative diagnostic odds ratio (RDOR) for cancer of 1.7 (95% CI 1.5-2.1, p < 0.001). Bleeding gave an AUC for cancer of 0.63 compared with 0.55 for polyps. For polyps alone, the DOR was 1.3 (95% CI 1.0-1.8; p = 0.08).

### Type of bleeding

Few papers provided information about bleeding type (details provided in Additional File 4) [8,19,31,34,41,48,49,59,62]. Of these, only bleeding mixed with stool and dark red blood had DORs consistently above 1. For bleeding mixed with stool the summary DOR was 3.1 (95% CI 2.0 - 4.8, p < 0.001), and the AUC 0.68. For dark red blood, the DOR was 3.9 (95% CI 1.7 - 9.2, p < 0.01), and the AUC was 0.71. No DOR and AUC was estimated for bright red bleeding, because this was reported in only 3 papers, for which the odds ratios were 0.9, 1.1 and 1.1 (Additional File 4).

### Weight loss

Eighteen papers provided information about the relationship between weight loss and colorectal cancer [8,18,19,22,25,26,32,34,35,37,47-49,55,59,61-63], and six of these papers [26,47-49,61,63] also provided information about the relationship between weight loss and polyps.

**Figure 3:**
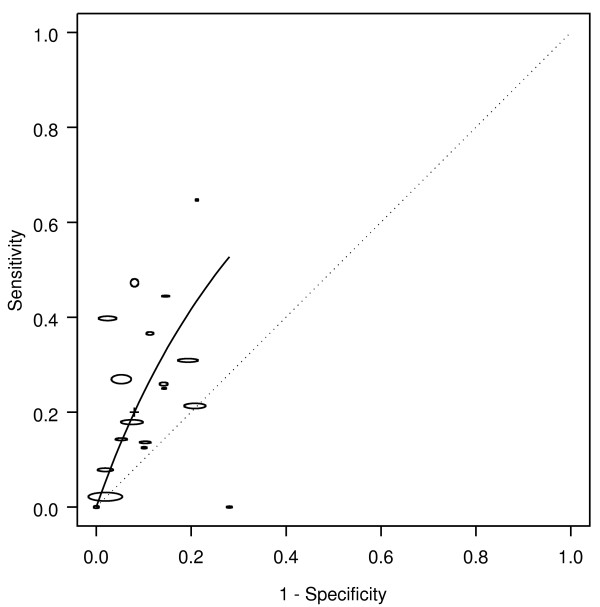
**Weight loss: SROC for cancer**. SROC = Summary ROC. crc = colorectal cancer. The horizontal and vertical dimensions of the each ellipse are proportional to the square root of the number of non-diseased and diseased respectively. + shows the expected sensitivity and 1-specificity for the curve. The diagonal line joining (0,0) and (1,1) represents an ROC of no diagnostic value.

Weight loss was associated with colorectal cancer, with an AUC of 0.67, corresponding to a DOR of 2.9 (Table 1 Figure 3). Weight loss occurred in 20% of the patients with cancer (sensitivity 0.20), and occurred in less than 10% without cancer (1-specificity 0.08). Hence, the likelihood of cancer was more than doubled in people presenting with weight loss (LR+ = 2.49). The corresponding likelihood of cancer in people presenting with no weight loss was LR- = 0.9.

**Figure 4:**
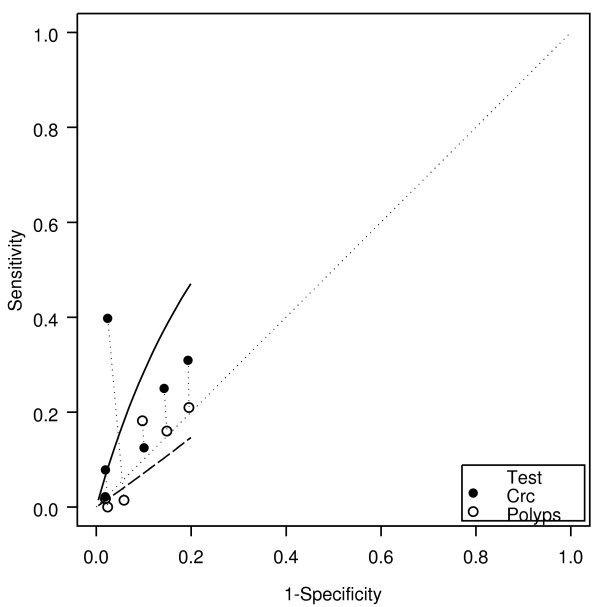
**Weight loss: cancer and polyp comparison**. crc = colorectal cancer. The horizontal and vertical dimensions of the each ellipse are proportional to the square root of the number of non-diseased and diseased respectively. + shows the expected sensitivity and 1-specificity for the curve. The paired points (one black and one open joined by a dotted line) represent a within study comparison. The diagonal line joining (0,0) and (1,1) represents an ROC of no diagnostic value.

Of the 6 "paired" studies that provided information about weight loss for both cancers and polyps, 5 had a higher DOR for cancer than for polyps (Figure 4), resulting in a RDOR of 5.2 (95% CI 2.8 - 9.6, p < 0.001). Weight loss showed better discrimination for cancer (AUC 0.70) than for polyps (AUC 0.44). Weight loss did not have a significant association with polyps (DOR 0.7, 95% CI 0.3 - 1.5, p = 0.22).

**Figure 5:**
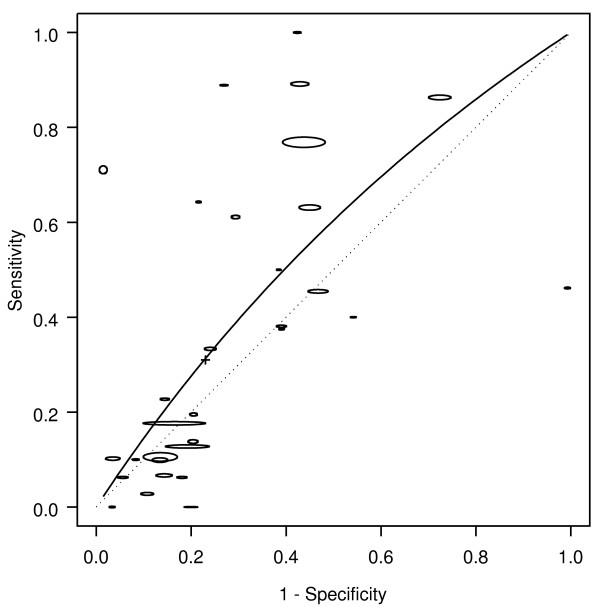
**Change in bowel habit: SROC for cancer**. SROC = Summary ROC. crc = colorectal cancer. The horizontal and vertical dimensions of the each ellipse are proportional to the square root of the number of non-diseased and diseased respectively. + shows the expected sensitivity and 1-specificity for the curve. The diagonal line joining (0,0) and (1,1) represents an ROC of no diagnostic value.

**Figure 6:**
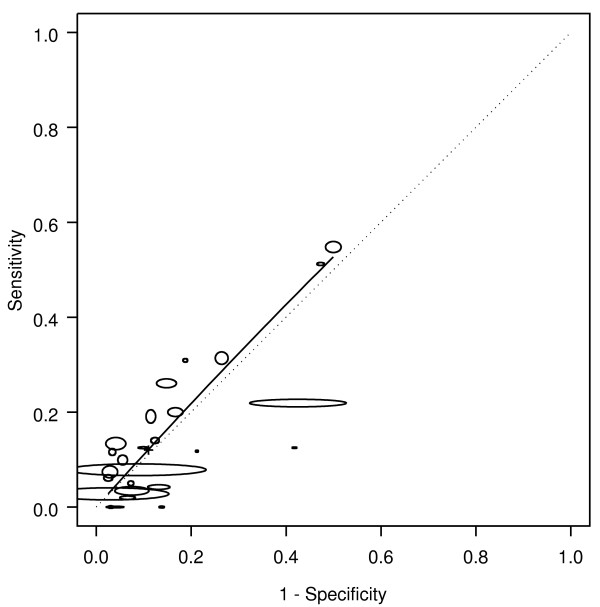
**Constipation: SROC for cancer**. SROC = Summary ROC. crc = colorectal cancer. The horizontal and vertical dimensions of the each ellipse are proportional to the square root of the number of non-diseased and diseased respectively. + shows the expected sensitivity and 1-specificity for the curve. The diagonal line joining (0,0) and (1,1) represents an ROC of no diagnostic value.

**Figure 7:**
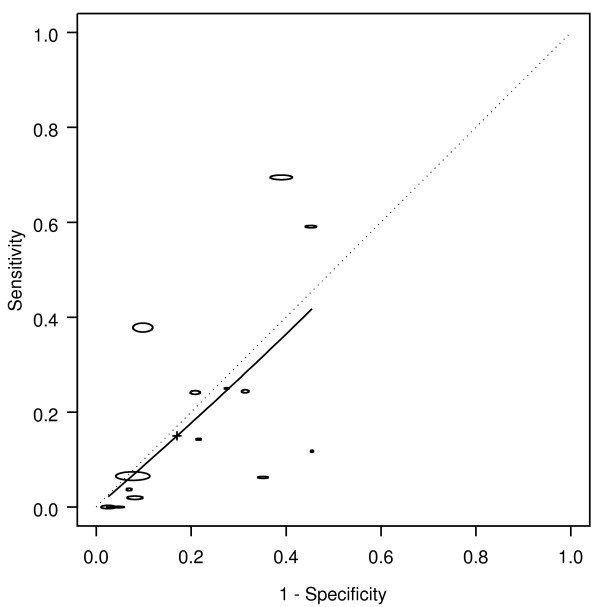
**Diarrhoea: SROC for cancer**. SROC = Summary ROC. crc = colorectal cancer. The horizontal and vertical dimensions of the each ellipse are proportional to the square root of the number of non-diseased and diseased respectively. + shows the expected sensitivity and 1-specificity for the curve. The diagonal line joining (0,0) and (1,1) represents an ROC of no diagnostic value.

**Figure 8:**
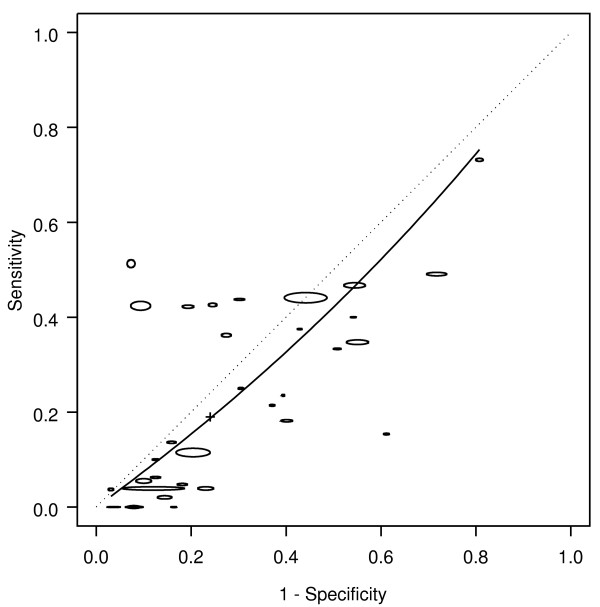
**Abdominal pain: SROC for cancer**. SROC = Summary ROC. crc = colorectal cancer. The horizontal and vertical dimensions of the each ellipse are proportional to the square root of the number of non-diseased and diseased respectively. + shows the expected sensitivity and 1-specificity for the curve. The diagonal line joining (0,0) and (1,1) represents an ROC of no diagnostic value.

Change in bowel habit, constipation, diarrhoea and abdominal pain were not associated with colorectal cancer (Table 1 Figure 5, Figure 6, Figure 7, Figure 8).

## Discussion

### Principal findings

The symptoms usually considered important for colorectal cancer diagnosis are rectal bleeding, change in bowel habit, abdominal pain, weight loss, diarrhoea and constipation. Of these, in our systematic review, only weight loss and rectal bleeding were associated with colorectal cancer, albeit with low DORs and AUCs. There was evidence that the other symptoms were not associated with colorectal cancer. Accuracy did not vary by primary care or specialist setting, or study type, suggesting that our findings are generaliseable across settings.

The lack of clinical usefulness of symptoms is also confirmed by the positive likelihood ratio of the symptoms. To provide strong evidence for ruling in disease, a positive likelihood ratio should be greater than 10 [74]. Faecal occult blood tests have been shown to have positive likelihood ratios of up to 47.39 [75]. Weight loss was the symptom with the highest positive likelihood ratio: 2.5. This means that a person with weight loss has less than a 3-fold increase in colorectal cancer risk. However, weight loss is generally a non-specific symptom, and in most of the studies included in this meta-analysis was analysed in a population already selected for being of sufficiently high risk of colorectal cancer to warrant investigation for colorectal cancer. Apart from weight loss and rectal bleeding, the positive likelihood ratio of other symptoms was around one. Our review also suggests that, even when symptoms are associated with cancer, they are not associated with polyps.

Previous systematic reviews of symptoms also found that clinical features have limited predictive value in identifying patients with cancer [76,77], although a systematic review of rectal bleeding in combination with other symptoms showed that this had modest diagnostic value [78]. Another review, assessing only those symptoms with a predictive value of > 5%, has also identified that some symptoms may be of some value [79]. Our review is based on a larger number of studies, evaluated both cancer and polyps, applied a more rigorous methodology, using hierarchical summary ROC method that takes into account unexplained heterogeneity between studies, and included assessment of whether study design or population characteristics affected the results.

### Quality of studies

Our review showed that most studies had methodological flaws. For example, the reference standard differed between studies and fewer than half the papers used colonoscopy to identify cancer and polyps. We have shown that the studies that used colonoscopy showed a somewhat stronger association between rectal bleeding and cancer (AUC 0.68) than when other methods were used to determine the presence of cancer (AUC 0.63). This is presumably because the association is underestimated when using a poorer reference standard [80]. However, most other measures of study quality were not shown to affect the results. Nevertheless, the quality of studies and their reporting should be improved. For example, we found that in over half the papers there were data discrepancies or miscalculations within the papers. While most of these were minor, 10% required some assumptions to extract the data. Adherence to quality criteria such as the STARD checklist and flow diagram would ensure that the quality of reporting [81].

Symptom presentation, and their predictive value, may be affected by the patient's age. However, the papers analysed did not present symptom and outcome information in sufficient detail to explore this relationship.

There was also no consistency between studies in the way in which symptoms were elicited or interpreted. Indeed very few studies provided information about how symptoms were elicited and did not characterise them: for example while some studies defined rectal bleeding as the first episode of bleeding or gave a time limit during which the bleeding occurred, the majority of studies provided no definition at all. Similarly, few studies gave definitions for constipation or diarrhoea or differentiated this from change in bowel habit. Also, there is potential for recall bias, with few studies providing information about when the symptoms were elicited in relation to when the diagnosis was made. In some studies symptoms were obtained from medical records, in other studies they were recorded during a consultation. In other studies a specific questionnaire was used, sometimes administered as part of the medical consultation and in others administered either by a researcher specifically for the study, or completed by the patients themselves. To do better studies in the future, a standardised repeatable method of eliciting symptoms is needed and is now available [82]. In practice symptoms are seldom assessed in isolation, and need to be evaluated together as well as with other patient characteristics. Where this has been done, it has been found that some additional symptoms may improve diagnostic value [78]. However, few studies have presented such information, and there is a need for a well conducted primary study to evaluate this.

## Conclusions

Our systematic review has shown that, on current evidence, only rectal bleeding and the general symptom of weight loss are associated with colorectal cancer, and may be helpful in selecting patients for further investigation with colonoscopy. Until such time as better studies are done, it seems wise to channel resources for cancer detection towards population based screening programmes using FOBT rather than relying on identifying all cancers and precancerous polyps through investigating people with symptoms.

## Abbreviations

AUC: area under the curve; crc: colorectal cancer.; DOR: diagnostic odds ratio; FOBT: faecal occult blood test; LR+: positive likelihood ratio; LR-: negative likelihood ratio; ROC: receiver operating characteristic; RDOR: relative receiver operating characteristic; SROC: Summary receiver operating characteristic; HSROC: hierarchical summary receiver operating characteristic; STARD: Standards for the reporting of diagnostic accuracy studies

## Competing interests

The authors declare that they have no competing interests.

## Authors' contributions

BA and LI: conceived the study; BA and LI reviewed the papers and extracted data; SC, PM and BA did the statistical analysis; All authors contributed to the interpretation of the data, ideas and the writing. All authors have read and approved the final manuscript.

## Authors' information

Barbara-Ann Adelstein, senior research fellow, Prince of Wales Clinical School, Faculty of Medicine, University of NSW, Sydney, Australia

Les Irwig, professor of epidemiology, STEP, School of Public Health, University of Sydney, Australia

Petra Macaskill, associate professor, STEP, School of Public Health, University of Sydney, Australia

Siew F Chan, biostatistician, STEP, School of Public Health, University of Sydney, Australia

Peter H Katelaris, gastroenterologist, Concord Repatriation General Hospital, University of Sydney, Australia

## Funding

This study was funded by the Australian National Health and Medical Research Council Program, grant 402764. All of the researchers involved in this project are independent of the funding body (Australian National Health and Medical Research Council) and the research had no industry sponsorship. We have not used any professional medical writers.

## Pre-publication history

The pre-publication history for this paper can be accessed here:

http://www.biomedcentral.com/1471-230X/11/65/prepub

## Supplementary Material

Additional file 1 Full search strategy (medline) Click here for file

Additional file 2 data categories and the assumptions required to extract the data Click here for file

Additional file 3 Characteristics of studies of symptoms and colorectal cancer or polyps Click here for file

Additional file 4 Bleeding type: association with cancer: DOR with 95% confidence intervals Click here for file
