# Supplementary material for: Most bowel cancer symptoms do not indicate colorectal cancer and polyps: a systematic review
Source: BMC Gastroenterol. 2011 May 30;11:65. doi: 10.1186/1471-230X-11-65 (PMC3120795; doi:10.1186/1471-230X-11-65)
Supplement: Additional file 1 — Full search strategy (medline) [file 1471-230X-11-65-S1.DOCX]

**Additional File 1: Full literature search terms**

1. *Abdominal Pain/co, di, et [Complications, Diagnosis, Etiology]

2. *Fatigue/co, di, et [Complications, Diagnosis, Etiology]

3. *Gastrointestinal Hemorrhage/co, di, et [Complications,Diagnosis, Etiology]

4. *Anemia, Hypochromic/co, di, et [Complications, Diagnosis, Etiology]

5. *Intestinal Obstruction/co, di, et [Complications, Diagnosis, Etiology]

6. *Constipation/co, di, et [Complications, Diagnosis, Etiology]

7. *Diarrhea/co, di, et [Complications, Diagnosis, Etiology]

8. *Anorexia/co, di, et [Complications, Diagnosis, Etiology]

9. *nausea/co, di, et [Complications, Diagnosis, Etiology]

10. *Dyspepsia/co, di, et [Complications, Diagnosis, Etiology]

11. *Flatulence/co, di, et [Complications, Diagnosis, Etiology]

12. Symptom$.mp.

13. *Weight Loss/

14. 1 or 2 or 3 or 4 or 5 or 6 or 7 or 8 or 9 or 10 or 11 or 12 or 13

15. exp Abdominal Pain/co, di, et [Complications, Diagnosis, Etiology]

16. exp Fatigue/co, di, et [Complications, Diagnosis, Etiology]

17. exp Gastrointestinal Hemorrhage/co, di, et [Complications,Diagnosis, Etiology]

18. exp Anemia, Hypochromic/co, di, et [Complications, Diagnosis, Etiology]

19. exp Intestinal Obstruction/co, di, et [Complications, Diagnosis, Etiology]

20. exp Constipation/co, di, et [Complications, Diagnosis, Etiology]

21. exp Diarrhea/co, di, et [Complications, Diagnosis, Etiology]

22. exp Anorexia/co, di, et [Complications, Diagnosis, Etiology]

23. exp nausea/co, di, et [Complications, Diagnosis, Etiology]

24. exp Dyspepsia/co, di, et [Complications, Diagnosis, Etiology]

25. exp Flatulence/co, di, et [Complications, Diagnosis, Etiology]

26. Symptom$.mp.

27. exp Weight Loss/

28. 15 or 16 or 17 or 18 or 19 or 20 or 21 or 22 or 23 or 24 or 25 or 26 or 27

29. Abdominal Pain/co, di, et [Complications, Diagnosis, Etiology]

30. FATIGUE/co, di, et [Complications, Diagnosis, Etiology]

31. Gastrointestinal Hemorrhage/co, di, et [Complications,Diagnosis, Etiology]

32. Anemia, Hypochromic/co, di, et [Complications, Diagnosis, Etiology]

33. Intestinal Obstruction/co, di, et [Complications, Diagnosis, Etiology]

34. Constipation/co, di, et [Complications, Diagnosis, Etiology]

35. Diarrhea/co, di, et [Complications, Diagnosis, Etiology]

36. Anorexia/co, di, et [Complications, Diagnosis, Etiology]

37. Nausea/co, di, et [Complications, Diagnosis, Etiology]

38. Dyspepsia/co, di, et [Complications, Diagnosis, Etiology]

39. Flatulence/co, di, et [Complications, Diagnosis, Etiology]

40. Symptom$.mp.

41. Weight Loss/

42. 29 or 30 or 31 or 32 or 33 or 34 or 35 or 36 or 37 or 38 or 39 or 40 or 41

43. exp *Abdominal Pain/co, di, et [Complications, Diagnosis, Etiology]

44. exp *Fatigue/co, di, et [Complications, Diagnosis, Etiology]

45. exp *Gastrointestinal Hemorrhage/co, di, et [Complications,Diagnosis, Etiology]

46. exp *Anemia, Hypochromic/co, di, et [Complications, Diagnosis, Etiology]

47. exp *Intestinal Obstruction/co, di, et [Complications, Diagnosis, Etiology]

48. exp *Constipation/co, di, et [Complications, Diagnosis, Etiology]

49. exp *Diarrhea/co, di, et [Complications, Diagnosis, Etiology]

50. exp *Anorexia/co, di, et [Complications, Diagnosis, Etiology]

51. exp *nausea/co, di, et [Complications, Diagnosis, Etiology]

52. exp *Dyspepsia/co, di, et [Complications, Diagnosis, Etiology]

53. exp *Flatulence/co, di, et [Complications, Diagnosis, Etiology]

54. Symptom$.mp.

55. exp *Weight Loss/

56. 43 or 44 or 45 or 46 or 47 or 48 or 49 or 50 or 51 or 52 or 53 or 54 or 55

57. exp Colorectal Neoplasms/pp, co, di, et [Physiopathology, Complications, Diagnosis, Etiology]

58. exp Rectal Diseases/pp, co, di, et [Physiopathology,Complications,Diagnosis, Etiology]

59. exp Colonic Diseases/pp, co, di, et [Physiopathology,Complications,Diagnosis, Etiology]

60. 57 or 58 or 59

61. exp *Colorectal Neoplasms/pp, co, di, et [Physiopathology, Complications, Diagnosis, Etiology]

62. exp *Rectal Diseases/pp, co, di, et [Physiopathology,Complications,Diagnosis, Etiology]

63. exp *Colonic Diseases/pp, co, di, et [Physiopathology,Complications,Diagnosis, Etiology]

64. 61 or 62 or 63

65. 14 and 60

66. 14 and 64

67. 28 and 60

68. 28 and 64

69. 42 and 60

70. 42 and 64

71. 56 and 60

72. 56 and 64

73. 65 and 66

74. 67 and 68

75. 69 and 70

76.71 and 72

77.73 or 74 or 75 or 76
