# Supplementary material for: Most bowel cancer symptoms do not indicate colorectal cancer and polyps: a systematic review
Source: BMC Gastroenterol. 2011 May 30;11:65. doi: 10.1186/1471-230X-11-65 (PMC3120795; doi:10.1186/1471-230X-11-65)
Supplement: Additional file 2 — data categories and the assumptions required to extract the data [file 1471-230X-11-65-S2.DOCX]

***Additional File 2: Data extraction: data extracted, with definitions and categories used for analysis***

| **Study Descriptors** | **Data Extraction: Assumptions made/definitions used** | **Details of papers** |
| --- | --- | --- |
| Year of publication | The years in which the papers in the systematic review were published extended from 1960 to 2005. We categorised the year of publication into 3 categories: before 1990; 1991-2000, and after 2000. | \| **Publication year** \| **Papers (n)** \| \| --- \| --- \| \| Up to 1990 \| 13 \| \| 1991-2000 \| 22 \| \| From 2001 \| 27 \| |
| Prospective or retrospective | We defined studies as **prospective** if patients were recruited after the study criteria were defined.  We defined studies as **retrospective** if the data was collected from data already available and collected for other purposes. This included sources such as medical records or databases. | \|  \| **Papers (n)** \| \| --- \| --- \| \| Prospective \| 39 \| \| Unknown (includes case control studies) \| 23 \| |
| Information source | We assessed how the symptom information used in the study was elicited. We grouped these into whether or not the information was collected purposely for the study. Information collected purposely for the study was by questionnaire (administered by a clinician or completed by the patient), or from a history and clinical examination done as part of the study. Information not collected purposely was taken from the patients’ medical record. | \| **Summary** \| **Papers (n)** \| \| --- \| --- \| \| Purposely collected \| 46 \| \| \| Not purposely collected \| 13 \| \| Not stated \| 3 \| |

*Table continued on next page*

**Appendix 2: Data extraction: data extracted, with definitions and categories used for analysis (continued)**

| **Study Descriptors** | **Data Extraction: Assumptions made/definitions used** | **Details of papers** |
| --- | --- | --- |
| Patient recruitment: consecutive or not? | In some studies it was not stated if the recruitment was consecutive or not. Where this occurred, if it was obvious form the design, the appropriate recruitment was recorded. However, in some papers this was not possible to identify, and ’not stated’ was recorded. Assumption made: if data was taken retrospectively from a database of endoscopy patients, it was assumed that consecutive patients were recruited. | \| **Summary** \| **Papers (n)** \| \| --- \| --- \| \| Yes \| 39 \| \| Unknown(includes case control studies \| 23 \| |
| Study type | Some studies were cross sectional in design, but had an additional component of follow up. These studies were classified as cross-sectional.  If there was doubt about a study type, the higher quality study type was chosen – this optimises results. | \| **Study Design** \| **Papers (n)** \| \| --- \| --- \| \| Cross sectional analytical \| 42 \| \| Case control \| 14 \| \| Cohort \| 5 \| \| Combined: cohort and case review \| 1 \| |
| Patient source: clinical setting of the study | The majority of patients were recruited or identified from endoscopy units or radiology centres. Data was extracted about the source of referral of these patients to these units. Patients were also recruited from other clinical settings. The clinical setting was summarised into those from general practice and those from a specialist setting. | \| **Summary** \| **Papers (n)** \| \| --- \| --- \| \| General practice (including screening, community) \| 19 \| \| Specialist \| 29 \| \| Mixed (case control) \| 14 \| |

**Appendix 2: Data extraction: data extracted, with definitions and categories used for analysis (continued)**

| **Study Descriptors** | **Data Extraction: Assumptions made/definitions used** | **Details of papers** |
| --- | --- | --- |
| Population type: Symptoms present in study population | In some studies patients in the study all had symptoms, while in others asymptomatic patients were also included. In addition, some papers presented information from a population of all bleeders: in these papers information was also provided about symptoms other than bleeding.  We grouped the papers into those where all the participants had symptoms and those in which asymptomatic people were also included. | \| **Symptom background** \| **Papers (n)** \| \| --- \| --- \| \| Included symptomatic and asymptomatic people \| 40 \| \| All FOBT +ve \| 1 \| \| All people had bleeding \| 7 \| \| All symptomatic (asymptomatic people symptoms not included) \| 15 \| |
| Number of symptoms reported per patient | Studies differed in how they reported the presence of symptoms. In some studies, patients could have more than one symptom/indication for colonoscopy recorded,(ie non-mutually exclusive) while in others only 1 symptom or indication was recorded (ie symptoms were mutually exclusive). The significance of this is that in non-mutually exclusive papers it is likely that all symptoms present were recorded, whereas in mutually exclusive papers it was possible (and indeed likely) that patients had additional symptoms that were not reported. In only 1 paper was the hierarchy for the reporting of symptoms recorded. | \| **Number of symptoms reported** \| **Papers (n)** \| \| --- \| --- \| \| Maximum of 1 per patient \| 19 \| \| Any number reported \| 43 \| |

*Table continued on next page*

**Appendix 2: Data extraction: data extracted, with definitions and categories used for analysis (continued)**

| **Study Descriptors** | **Data Extraction: Assumptions made/definitions used** | **Details of papers** |
| --- | --- | --- |
| Ease with which data relevant to the systematic review was available in paper | The quality of the data analysis and presentation varied in the papers; in some cases major assumptions about either the study or the figures given needed to be made. Data extraction was categorised as listed below, with the assumptions made to rectify problems identified:   - Easy (all information in the paper); - all information is in the paper, but with minor miscalculations (usually typographical errors) of numbers with rectification obvious; obvious corrections made - all information in the paper, but with minor miscalculations where rectification was not obvious; calculation made using other data provided – for eg discrepancy between data provided in a table and in the text: assumption made: use data in table, unless obviously incorrect; use table providing the most information, or most consistent information. - all information was in the paper, but data required needed to be recalculated (however, the results do not equal other values in paper – eg OR); - major assumptions were needed to extract figures: for example if the denominator is known, use maximum number of people participating in a study) - assumptions were needed about the methodology | \| **Data accessibility** \| **Papers (n)** \| \| --- \| --- \| \| Easy/minor issues \| 52 \| \| Major issues \| 10 \|   Note: There were 2 papers with 2 problems and 1 paper with 3 problems. |

*Table continued on next page*

**Appendix 2: Data extraction: data extracted, with definitions and categories used for analysis (continued)**

| **VALIDITY CRITERIA** | | |
| --- | --- | --- |
| Reference standard | Studies used a range of reference standards: colonoscopy, sigmoidoscopy (flexible or rigid), barium enema, clinical follow up or a combination of these. One study did not have a single reference standard, but used multiple diagnostic modalities.  4 studies were case control studies: cases were diagnosed by colonoscopy, but the controls had no verification | \| **Diagnostic test used** \| **Papers (n)** \| \| --- \| --- \| \| Colonoscopy \| 28 \| \| All others \| 34 \| |
