# Supplementary material for: Most bowel cancer symptoms do not indicate colorectal cancer and polyps: a systematic review
Source: BMC Gastroenterol. 2011 May 30;11:65. doi: 10.1186/1471-230X-11-65 (PMC3120795; doi:10.1186/1471-230X-11-65)
Supplement: Additional file 3 — Characteristics of studies of symptoms and colorectal cancer or polyps [file 1471-230X-11-65-S3.DOCX]

**Additional File 3: Characteristics of studies of symptoms and colorectal cancer or polyps**

| **Author** | **Year** | **Pop Type** | **Mut Excl** | **Design Type** | **age range** | **No in study** | **Cancer (n)** | **Polyp (n)** | **Bleeding** | **Abdo pain** | **CBH** | **Constip** | **Diarrhoea** | **Weight loss** | **Other** |
| --- | --- | --- | --- | --- | --- | --- | --- | --- | --- | --- | --- | --- | --- | --- | --- |
| **Adler** | **2007** | **S+N** | **1** | **xs** | **16-87** | **1,398** | **11** | **214** | **X** | **X** |  | **X** | **X** |  |  |
| **Ahmed** | 2005 | B | 2 | xs | 50-69 | 563 | 43 | 279 | X |  |  |  |  |  |  |
| **Bafandeh** | 2008 | S | 2 | xs |  | 480 | 16 | 56 | X | X | X | X | X |  | X |
| **Bat** | 1992 | S+N | 2 | xs | 80 -101 | 436 | 29 | 130 | X |  |  |  |  |  |  |
| **Berkowitz** | 1993 | S+N | 1 | xs | 15-93 | 448 | 16 | 59 | X | X | X |  |  |  |  |
| **Bhatti** | 2004 | S | 2 | xs | 40-70 | 50 | 17 |  | X | X |  | X | X | X | X |
| **Bjerregaard** | 2007 | S | 2 | xs | 40-97 | 2,172 | 122 |  | X | X | X |  |  | X | X |
| **Brenna** | 1990 | S+N | 1 | xs |  | 833 | 45 | 203 | X |  |  |  |  |  | X |
| **Brewster** | 1994 | S | 2 | xs |  | 462 | 21 | 60 | X | X | X |  |  |  |  |
| **Chak** | 1996 | S+N | 2 | xs | 21-99 | 653 | 28 |  |  |  |  |  |  | X | X |
| **Charalambopoulos** | 2000 | S | 1 | xs | 23-82 | 795 | 3 | 207 | X | X | X |  |  |  |  |
| **Cheong** | 2000 | S+N | 1 | xs | 13-92 | 375 | 22 | 53 | X | X | X |  |  |  | X |
| **Curless** | 1994 | S+N | 2 | cc | 20-99  (17 pts<40) | 546 | 273 |  | X | X | X |  |  | X | X |
| **de Bossett** | 2002 | S+N | 1 | xs | 16-95 | 1,144 | 51 | 104 | X | X |  | X | X | X | X |
| **Douek** | 1999 | S | 1 | xs |  | 455 | 64 |  | X |  |  |  |  |  |  |
| **Dukas** | 2000 | S+N | 2 | c |  | 84,438 | 611 |  |  |  |  | X |  |  |  |
| **Duncan** | 2006 | S+N | 1 | xs | 80+ | 1,199 | 45 | 108 | X |  | X |  |  |  | X |
| **du Toit** | 2006 | S+N | 2 | xs | 45-75 | 2,889 | 38 | 33 | X |  |  |  |  |  |  |
| **Ellis** | 2005 | B | 2 | xs | 35-89+ | 266 | 11 | 17 |  |  | X |  |  |  |  |
| **Farrands** | 1985 | S | 2 | xs | 30-80+ (majority 50-70) | 152 | 13 |  | X | X | X |  |  | X |  |
| **Ferraris** | 2004 | S+N | 1 | xs | 55-64 | 8,507 | 46 | 1,398 | X |  | X |  |  |  | X |
| **Fitjen** | 1995 | B | 2 | xs | 18 -75 | 269 | 9 |  |  | X | X |  |  | X | X |
| **Fontagnier** | 2000 | S+N | 1 | xs | 80-94 | 157 | 27 |  | X | X |  | X | X | X | X |
| **Haenszel** | 1973 | S+N | 2 | cc |  | 536 | 179 |  |  |  |  | X |  |  |  |
| **Hamilton** | 2005 | S+N | 2 | cc |  | 2,093 | 349 |  | X | X |  | X | X | X | X |
| **Higginson** | 1966 | S+N | 2 | cc |  | 1,360 | 340 |  |  |  |  | X |  |  |  |
| **Jacobs** | 1998 | S+N | 2 | cc | 30-62 | 838 | 424 |  |  |  |  | X |  |  |  |
| **Jain** | 1980 | S+N | 2 | cc |  | 1619 | 542 |  |  |  |  | X |  |  |  |
| **Jensen** | 1993 | S | 2 | xs | 52 – 74 | 149 | 5 | 10 | X | X | X |  |  |  | X |
| **Kassa** | 1996 | S+N | 1 | sx | 11 – 83 | 640 | 45 | 59 | X | X | X |  |  |  | X |
| **Kojima** | 2004 | S+N | 2 | c |  | 62,929 | 649 |  |  |  |  | X |  |  |  |
| **Kune** | 1988 | S+N | 2 | cc |  | 1408 | 685 |  |  |  |  | X |  |  |  |
| **Lee** | 2002 | S | 2 | xs | 14-91 | 869 | 43 |  | X |  |  |  |  |  |  |
| **Leis** | 2001 | S+N | 2 | xs | 28-85 | 202 | 10 | 108 | X | X | X |  |  |  | X |
| **Leung** | 2006 | S | 1 | xs | 65%>60yrs | 5,464 | 322 | 512 | X | X | X | X | X | X | X |
| **Mant** | 1989 | B | 2 | c | 40 – 95 | 144 | 16 | 11 |  | X | X |  |  | X | X |
| **Metcalf** | 1996 | B | 2 | xs | 40 -86 | 99 | 8 | 25 |  | X | X | X | X | X |  |
| **Morini** | 2001 | S+N | 1 | xs |  | 966 | 49 |  | X | X | X |  | X |  | X |
| **Nakama** | 2000 | S+N | 2 | xs | 40-60+ | 9,625 | 31 |  | X |  |  |  |  |  |  |
| **Nakamura** | 1984 | S+N | 2 | cc |  | 251 | 100 |  |  |  |  | X |  |  |  |
| **Nascimbeni** | 2002 | S+N | 1 | cc | 39-95 | 151 | 55 |  |  |  |  | X |  |  |  |
| **Neugent** | 1993 | S+N | 2 | xs | 35-84 | 1,172 | 91 | 275 | X | X | X |  |  |  | X |
| **Norrelund** | 1996 | B | 2 | c |  | 364 | 54 |  |  | X | X |  |  | X | X |
| **Panzuto** | 2003 | S+N | 2 | xs | 18-87 | 280 | 41 |  | X | X | X | X | X | X | X |
| **Park** | 2006 | S+N | 1 | xs | 21-78 | 17,307 | 51 | 4136 | X | X | X |  |  |  | X |
| **Pernu** | 1960 | S+N | 2 | cc |  | 2,439 | 666 |  |  |  |  | X |  |  |  |
| **Roberts** | 2003 | S+N | 1 | cc |  | 1,675 | 634 |  |  |  |  | X |  |  |  |
| **Robertson** | 2006 | B | 2 | xs | 18-97 | 604 | 22 |  |  | X |  |  | X | X | X |
| **Sardinha** | 1999 | S+N | 1 | xs | 80-95 | 428 | 10 |  | X |  |  |  |  |  |  |
| **Schoepfer** | 2005 | S | 2 | xs | 50-80 | 1,514 | 83 | 484 | X |  | X |  |  | X | X |
| **Selvachandran** | 2002 | S | 2 | xs |  | 2,268 | 95 |  | X | X | X | X | X | X | X |
| **Steine** | 1994 | S+N | 2 | xs |  | 1,852 | 55 | 186 | X | X | X |  |  | X | X |
| **Tan** | 2002 | S+N | 2 | xs |  | 485 | 58 |  | X | X | X |  | X |  | X |
| **Tate** | 1988 | S | 2 | xs | 20-83 | 130 | 14 |  | X | X | X | X | X |  | X |
| **Thompson** | 2007 | S | 2 | xs | 52%> 60yrs | 8,529 | 467 |  | X | X | X |  |  |  | X |
| **Vobecky** | 1983 | S+N | 2 | cc |  | 414 | 207 |  |  |  |  | X |  |  |  |
| **Watanabe** | 2004 | S+N | 2 | c |  | 41,670 | 251 |  |  |  |  | X |  |  |  |
| **Wauters** | 2000 | S | 2 | combined |  | 7,886 | 106 |  | X |  |  |  |  |  |  |
| **Wynder** | 1967 | S+N | 2 | cc |  | 1,200 | 791 |  |  |  |  | X |  |  |  |
| **Wynder** | 1969 | S+N | 2 | cc |  | 464 | 157 |  |  |  |  | X |  |  |  |
| **Zbar** | 1999 | S+N | 1 | xs | 19-85 | 744 | 36 | 86 | X | X | X |  |  |  | X |
| **Zerey** | 2007 | S+N | 1 | xs | 85-99 | 157 | 8 | 108 | X | X | X |  |  |  | X |
| **TOTAL** |  |  |  |  |  |  |  |  | 40 | 33 | 31 | 26 | 14 | 18 | 32 |

Notes:

Pop type = population from which participants drawn: S=people with and with no symptoms N=all people had symptoms B=all had bleeding

Mut excl = mutually exclusive; refers to the number of symptoms that could be reported, ie whether or not the symptoms were mutually exclusive: 1= only 1 symptom per participant 2= any number of symptoms could be reported for each participant

Design Type: xs = cross sectional analytical; cc= case control; c= cohort

Abdo pain = abdominal pain; Constip = constipation CBH =Change in bowel habit X= reported
