# Supplementary material for: Most bowel cancer symptoms do not indicate colorectal cancer and polyps: a systematic review
Source: BMC Gastroenterol. 2011 May 30;11:65. doi: 10.1186/1471-230X-11-65 (PMC3120795; doi:10.1186/1471-230X-11-65)
Supplement: Additional file 4 — Bleeding type: association with cancer: DOR with 95% confidence intervals [file 1471-230X-11-65-S4.DOCX]

**Additional File 4 Bleeding type: association with cancer: DOR with 95% confidence intervals**

| **Paper (Author)** | **Mixed with stool** | **On toilet paper** | **Colours water** | **On paper and in bowl** | **Separate from stool** | **Bright red** | **Dark red** | **First episode** | **Large (vs small) volume** | **First episode (vs changed)** |
| --- | --- | --- | --- | --- | --- | --- | --- | --- | --- | --- |
| **Bjerregaard (2007)** |  |  |  |  |  | 0.9  (0.6-1.3) | 5.6  (3.6- 8.8) |  |  |  |
| **Ellis (2005)** | 1.0  (0.2-5.7) | 0.6  (0.1-2.4) |  |  |  |  | 2.8  (0.7-10.2) | 1.3  (0.4-4.1) | 0.3  (0.1-1.8) |  |
| **Fitjen (1995)** | 3.4  (0.9-12.3) |  |  |  |  |  |  |  |  |  |
| **Jensen (1993)** | 3.2  (0.6-17.1) | 0.4  (0.0-6.6) |  |  |  |  |  |  |  |  |
| **Mant (1989)** | 2.6  (0.9-7.2) | 0.8  (0.3-2.2) | 1.4  (0.4-4.9) | 0.9  (0.3-2.6) | 0.2  (0.1-0.6) |  |  |  |  |  |
| **Metcalf (1996)** | 1.9  (0.5-7.8) | 1.2  (0.3-5.5) |  |  |  | 1.1  (0.2-5.1) | 1.4  (0.3-5.8) |  |  |  |
| **Norrelund (1996)** |  |  |  |  |  |  |  |  |  | 0.7  (0.3-1.5) |
| **Robertson (2006)** | 3.1  (1.2-8.1) |  |  |  |  |  | 4.5  (1.9-10.6) |  |  |  |
| **Selvachandran (2002)** | 3.7  (2.3-5.8) |  |  | 2.1  (1.2-4.0) | 0.8  (0.5-1.2) | 1.1  (0.7-1.6) | 3.0  (1.4-6.6) |  |  |  |
